# Synthesis of Silver Particle onto Bamboo Charcoal by Tripropylene Glycol and the Composites Characterization

**DOI:** 10.3390/ma7020742

**Published:** 2014-01-27

**Authors:** Tzu Hsuan Chiang, Hung Che Yeh

**Affiliations:** Department of Energy Engineering, National United University, Miaoli 36003, Taiwan; E-Mail: kevin25831@yahoo.com.tw

**Keywords:** silver partilcle, bamboo charcoal, tripropylene gycol, thermal conductivity

## Abstract

In this study, tripropylene glycol was used as a reducting agent in the polyol process to reduce silver nitrate to the form of silver particles deposited onto the surface of bamboo charcoal (BC). The reduction temperature and time were critical parameters as they control the size of the silver particles formed as well as their distribution onto the surface of the BC. The reduction of silver nitrate by the tripropylene glycol occurred at a temperature of 120 °C for 3 h, and the silver particles, which had a face-centered cubic lattice structure, were distributed onto the surface of the BC. These synthesis conditions should work well with tripropylene glycol as reducing agent that can be helpful in the convenient preparation of Ag/BC particles. When Ag/BC powders were manufactured using 3 g of silver nitrate content, the prepared composites had the largest thermal conductivity at 0.2490 W/(m·K).

## 1. Introduction

Bamboo charcoal (BC) powder is often used to make BC fibers used extensively in making clothes, socks, and towels in Taiwan and China. Because these BC fibers maintain their functional ability as effective adsorption media, they eliminate odors, improve air quality, exhibit bacteriostatic properties, block electromagnetic radiation, and regulate temperature and humidity effects [1].

Some studies have investigated the use of silver-coated fibers, such as silver-coated nylon fibers, as an anti-bacterial agent that can be manufactured by an electrodeless plating process [2]. Such anti-microbial fibers have been produced by placing nanoscale silver particles into a solution of cellulose and N-methylmorpholine-N-oxide (NMMO) [3]. In addition, silver-coated carbon fibers that possess anti-microbial properties have been prepared using a dynamic method [4], and ALCERU^®^ silver fibers [3,5] are used in textile applications in which products are manufactured by combining these fibers with other fibers to yield yarns. Yang *et al.* [6] prepared BC-supporting silver (BC/Ag) with antibacterial properties using ammonium hydroxide and chemical reduction.

To synthesize silver particles, most studies have used ethylene glycol as the reducing agent; however, compared to our approach, these synthesis methods have required higher temperatures and more time (e.g., the reactions occurred at temperatures ranging from 120 to 186 °C, requiring 4–22 h to synthesize the Ag particles [7,8]). Therefore, in this study, we used tripropylene glycol monomer as the reducing agent to form silver particles on the BC at reaction temperatures as low as 100 °C for a time of only 2 h. Depositing silver particles onto a BC surface using tripropylene glycol in the polyol process has not yet been studied.

The current study uses tripropylene glycol to synthesize silver particles on a BC surface in order to study the effects of reducing the temperature and reaction time on the sizes morphologies of Ag/BC particles. Relevant discussions related to the thermal conductivity and electrical conductivity are presented as well.

## 2. Results and Discussion

### 2.1. Effect of Reduction Temperature

**Figure 1.**
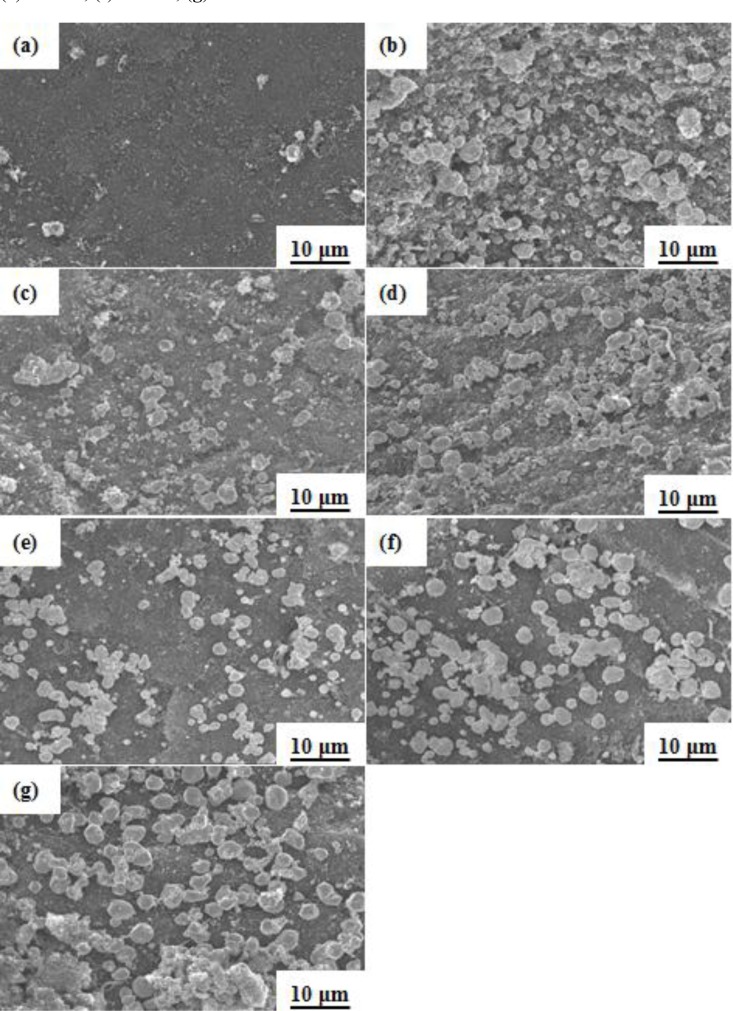
Scanning electron microscopy (SEM) images of Ag/bamboo charcoal (BC) for different reduction temperatures for 2 h: (**a**) blank; (**b**) 100 °C; (**c**) 120 °C; (**d**) 130 °C; (**e**) 140 °C; (**f**) 150 °C; (**g**) 160 °C.

Reaction temperature was a crucial parameter in the synthesis of the silver particles onto BC particles. Figure 1a shows the surface of the BC. The silver particles were synthetized onto the surfaces of the BC particles by using reduction temperatures of 100, 120, 130, 140, 150 and 160 °C for 2 h, as shown in Figure 1b–e. The sizes of the silver particles in the BC powder increased from 0.5 to 4 μm as the reduction temperature increased—that is, the ranges were 0.5 to 1.5 ± 0.3 μm, 1.0 to 2.0 ± 0.2 μm, 2.0 to 3.0 ± 0.2 μm, and 3.0 to 4.0 ± 0.3 μm for reduction temperatures of 100 °C, 130–140 °C, 150 °C, and 160 °C, respectively. Increases in the reduction temperature resulted in increased reaction rates, thereby reducing the time required for the reaction to be completed. Thus, for the same reaction time, there was sufficient solute in contact with the growing crystals at the higher temperatures to increase the diameter of the silver particles on the surface of the BC.

According to the polyol reduction mechanism proposed by Fievet *et al.* [9], Ag is formed by the reduction of Ag^+^ ions via the polyol process by tripropylene glycol, as depicted below:

$$2{\text{HOC}}_{3}{\text{H}}_{6}-{\left({\text{OC}}_{3}\text{H}6\right)}_{2}-\text{OH}\;\to \;2{\text{OC}}_{3}{\text{H}}_{5}-{\left({\text{OC}}_{3}{\text{H}}_{6}\right)}_{2}\text{H}\;+\;2{\text{H}}_{2}\text{O}$$ (1)

$$2{\text{Ag}}^{+}+\;2{\text{OC}}_{3}{\text{H}}_{5}-{\left({\text{OC}}_{3}{\text{H}}_{6}\right)}_{2}\text{H}\;\to \;\text{H}{\left({\text{OC}}_{3}{\text{H}}_{6}\right)}_{2}-{\text{C}}_{3}{\text{H}}_{4}\text{O}-{\text{OC}}_{3}{\text{H}}_{4}{\left({\text{OC}}_{3}{\text{H}}_{6}\right)}_{2}\text{H}\;+\;2\text{Ag}\;+\;2{\text{H}}^{+}$$ (2)

**Figure 2.**
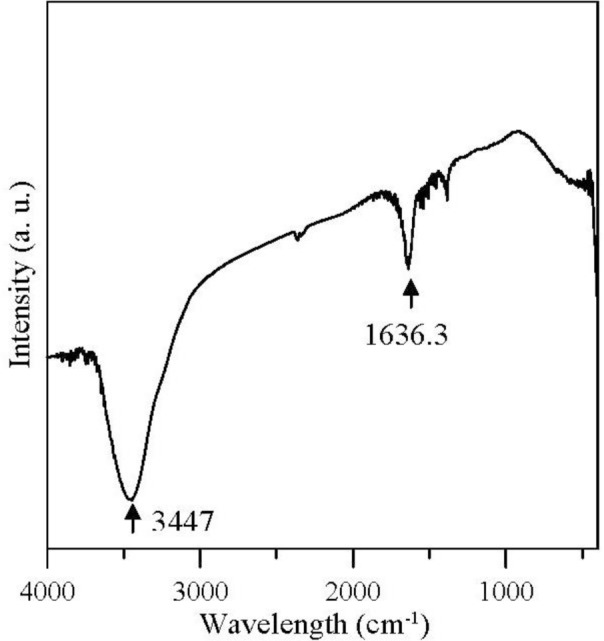
Fourier transform infrared (FTIR) spectra of Ag/BC particles.

Figure 2 shows that the FTIR spectra of the Ag/BC particles peaked at 1636.3 cm^−^^1^, indicating that the C=O that was complexed with silver salt [10,11] demonstrated a specific interaction between the carbonyl oxygen of the OC_3_H_5_-(OC_3_H_6_)_2_H [see Equation (1)] and the silver cations. The peak at 3447 cm^−^^1^ was due to the presence of H_2_O absorbed from the air.

**Figure 3.**
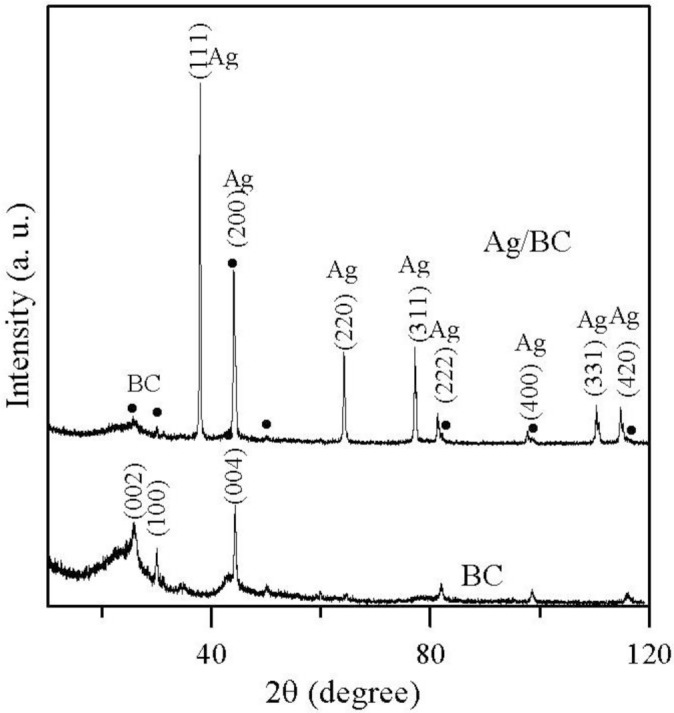
X-ray diffraction (XRD) pattern of Ag/BC and BC particles.

The samples prepared using the polyol process were analyzed using X-ray diffraction (XRD) to obtain a better understanding of the crystal structure and composition of the Ag particles on the Ag/BC composites. Figure 3 shows the XRD pattern of the as-prepared silver particles on the Ag/BC composites. The main peaks coincide with the 2θ values at 37.88°, 44.06°, 64.2°, 77.2°, 81.36°, 97.78°, 110.38°, and 114.9° that correspond to the (111), (200), (220), (311), (222) (400), (331), and (420) crystal planes of the face-centered cubic (fcc) lattice of silver (JCPDS card No. 04-0783), respectively [12]. They also possess different surface energies for different crystal planes. In addition, the peaks of the black circle in the Ag/BC curve represented the peaks of the BC particle.

### 2.2. Effect of Reduction Time

**Figure 4.**
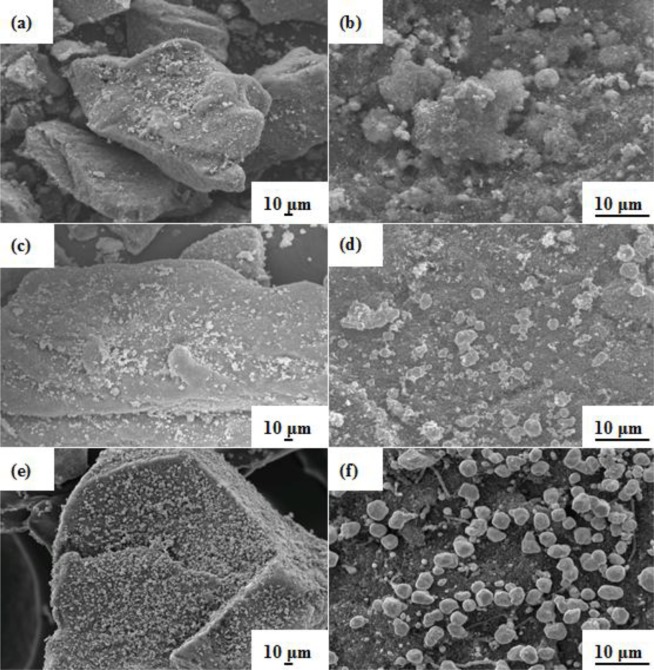

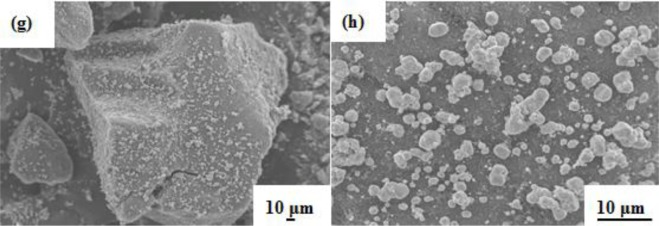
SEM images of silver particles on BC at 120 °C for various reduction times as (**a**) 0.5 X and (**b**) 2 X magnification of 1 h; (**c**) 0.5 X and (**d**) 2 X magnification of 2 h; (**e**) 0.5 X and (**f**) 2 X magnification of 3 h; (**g**) 0.5 X and (**h**) 2 X magnification of 4 h.

More uniformly sized silver particles formed at 120 °C than at 100 °C. Therefore, the particle size of silver was investigated using different reduction times at 120 °C. Fifty milliliters of tripropylene glycol, 1 g of AgNO_3_, and 2 g of BC powder were mixed and underwent reduction at 120 °C, with different reduction times. Figure 4 shows the SEM images of the Ag particles synthesized at different reduction times. The results indicated that silver particles with a range of sizes were formed and distributed onto the surfaces of the BC for reduction times of 1 and 2 h, as shown in Figure 4a–d. When the reduction time was increased to 3 h, silver particles were formed at sizes of about 3 μm, and the particles were distributed homogeneously on the surfaces of the BC, as shown in Figure 4e,f. At the reduction time of 4 h, the silver particles were beginning to aggregate, which caused the silver particles to have inhomogeneous sizes and distributions on the BC surfaces, as shown in Figure 4g,h. The results indicated that the reduction time should be 3 h, which provides appropriate numbers of available nuclei to induce the growth of the particles.

### 2.3. Effect of Different Content of Silver Nitrate

**Figure 5.**
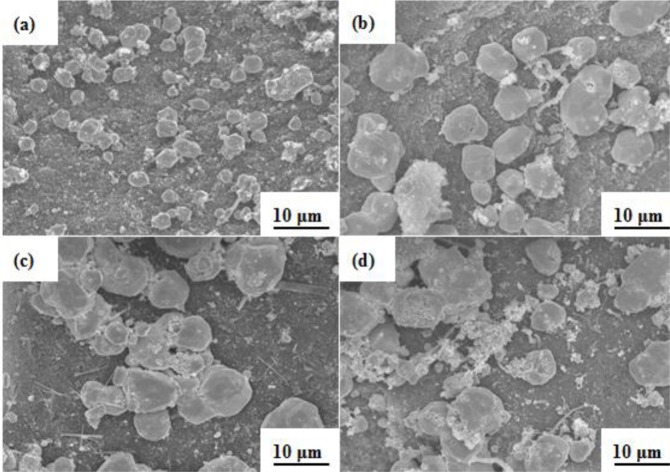
SEM images of silver particles on BC at 120 °C for various AgNO_3_ content as (**a**) 1 g; (**b**) 2 g; (**c**) 3 g; and (**d**) 4 g.

At 120 °C, 50 mL of tripropylene glycol, 2 g of BC, and different contents of silver nitrate (*i.e.*, 1 g, 2 g, 3 g, and 4 g) were stirred for 30 min, forming the SEM images shown in Figure 5. In the process, we found that the particle sizes of silver forming on the BC increased as the content of the silver nitrate increased because the collision frequency was increased, thereby causing the formation of more Ag atoms in the same conditions. When Ag/BC particles were prepared by 4 g of silver nitrate, more aggregate Ag particles were formed for each other, as shown in Figure 5d.

Table 1 indicates that the thermal conductivity and electrical resistivity of composites contain blank (12 wt% of BC) and 12 wt% of Ag/BC powder due to various silver nitrate preparations. The results show that the electrical resistivity of the blank was the lowest at 3.26 × 10^−^^4^ Ω·cm. However, the electrical resistivity increased as Ag/BC powder prepared by silver nitrate increased because the Ag particles of the Ag/BC powder had no coating as a preservation agent on the surface; thus, the Ag particles were easily oxidized in air, forming greater electrical resistivity. Therefore, Ag/BC particles were prepared using 4 g of silver nitrate, which showed the largest electrical resistivity at 0.326 Ω·cm. In addition, the blank sample had the lowest thermal conductivity at 0.1921 W/(m·K). When the Ag/BC powder was prepared, the silver nitrate content increased, which helped enhance the thermal conductivity of composites. The largest thermal conductivity was 0.2490 W/(m·K), when Ag/BC powders were manufactured using 3 g of silver nitrate content. However, the Ag/BC powders were manufactured with 4 g of silver nitrate content, which caused the thermal conductivity of composites to decrease because the non-uniform distribution of the silver particles on the BC surface (see Figure 5d) meant that the individual Ag/BC particles could not have much content.

## 3. Experimental

### 3.1. Materials

The carbonation temperature of the BC provided by B&N Technology, Inc. (Suzhou, China), was 1400 °C, and the average size was 1 mm. For purification, the plain bamboo was dispersed in 200 mL of concentrated nitric acid and refluxed for 4 h at 80 °C. The BC was subsequently filtered and washed with distilled water to remove the nitric acid, after which it was dried at 120 °C for 10 h. The silver nitrate (AgNO_3_) was supplied by Hwang Long Co., Ltd., Tainan, in Taiwan; the 97 wt% pure tripropylene glycol [HOC_3_H_6_-(OC_3_H_6_)_2_-OH] was purchased from Sigma-Aldrich Co., Ltd., (St. Louis, MO, USA) and the ethanol was purchased from Echo Chemical Co., Ltd.,(Miaoli, Taiwan).

### 3.2. Synthesis of Ag/BC Particles

The Ag/BC particles were synthesized using the polyol process. First, 1 g, 2 g, 3 g, and 4 g of AgNO_3_ were dissolved in 50 mL of tripropylene glycol using a magnetic stirrer for 10 min, respectively. Then, 2 g of purified BC were dispersed into the solution using a magnetic stirrer for 30 min. After the solution was heated at 100, 120, 130, 140, 150, and 160 °C for 2 h, respectively, AgNO_3_/BC particles were obtained. The suspension was cooled to room temperature, centrifuged, washed with absolute ethanol three times to remove the remaining tripropylene glycol and any soluble by-products, and dried in a vacuum oven at 100 °C for 6 h to remove the solvent from the particles.

### 3.3. Preparation of Ag/BC Composites

The composites based on epoxy resin were prepared by mixing the 12 wt% of Ag/BC powder with the resin (bisphenol-A, GY 260, Araldite, Taipei, Taiwan) and hardener (HY 956, Araldite, Taipei, Taiwan) at a ratio of 10:1 by weight. After being mixed, the composites were placed in a 70-mm diameter pan and cured for 2 h at120 °C.

### 3.4. Characteristics of Ag/BC Particles

The X-ray diffraction (XRD) pattern of the prepared Ag/BC powder was obtained using a Rigaku TTRAX III rotating anode diffractometer with a Ni-filtered Cu Kα radiation source (Tokyo, Japan). The phases were identified using files acquired from the Joint Committee on Powder Diffraction Standards (JCPDS). The morphology of the silver particles was investigated via scanning electron microscopy (SEM) (Jeol, JSM5600LV, Tokyo, Japan), and the particle size distributions were obtained by image analysis. Fourier transform infrared (FTIR) spectra were obtained with a JASCO FT/IR-470 plus spectrometer (Easton, USA) in the wavelength range from 400 to 4000 cm^−1^ and a resolution of 4 cm^−1^ with each spectrum.

### 3.5. Thermal and Electrical Conductivity of Ag/BC Composites

Thermal conductivity measurements were conducted according to the ASTM D5930 test method using an ISOMET 2114 thermal conductivity instrument at room temperature. The electrical resistivity of the Ag/BC composite (thickness of 154 μm) was determined using a four-point probe instrument manufactured by Ever Being International Corporation (Hsinchu, Taiwan). The electrical resistivity, ρ, was calculated as follows:

$$\rho =4.532t\frac{V}{I}$$

where *t* is the thickness of the film, *V* is the voltage, and *I* is the directly current (DC) electric current supplied by a power supply (Tektronix, DMM40506-1/2 Dight Precision Multimeter, Beaverton, OR, USA).

## 4. Conclusions

The results demonstrated that the tripropylene glycol monomer can be used successfully to reduce the silver nitrate to silver particles at 100 °C for 2 h, and a homogeneous distribution of the particles on the BC surface occurred, although the sizes of the silver particles were inhomogeneous. When the reduction temperature was 120 °C for 3 h, both the particle size and distribution of the BC were homogeneous, and a face-centered cubic lattice of silver particles was formed on the surface of the BC. As the reduction temperature and contents of silver nitrate used in the polyol process were increased, the diameter of the silver particles on the surface of the BC also increased. When Ag/BC powders were manufactured using 3 g of silver nitrate content, the prepared composites had the largest thermal conductivity at 0.2490 W/(m·K). The Ag particles of the Ag/BC powder were without any coating or preserving agent on the surface, meaning that the Ag particles were easily oxidized and the composites had greater electrical resistivity.

## Figures and Tables

**Table 1.** Thermal conductivity and electrical resistivity of Ag/BC powder of composites prepared using various amounts of silver nitrate.

Silver nitrate content (g)	Thermal conductivity [W/(m·K)]	Electrical resistivity (Ω·cm)
Blank	0.1921	3.26 × 10^−4^
1	0.2268	7.34 × 10^−4^
2	0.2314	1.79 × 10^−1^
3	0.2490	2.35 × 10^−1^
4	0.2277	3.26 × 10^−1^
